# Preliminary assessment of *Onchocerca*-induced visual impairment using clinical fundus camera in Gashaka local government area of Taraba state, north eastern Nigeria

**DOI:** 10.1016/j.parepi.2023.e00296

**Published:** 2023-03-11

**Authors:** Francisca O. Olamiju, Hammed O. Mogaji, Marcus Trappaud Bjørn, Ayodele J. Marcus, Vera Oduwa, Olatunwa J. Olamiju, Markus Nzunde, David K. Ikyerga, Adrian Hopkins

**Affiliations:** a Mission To Save The Helpless (MITOSATH), Jos, Nigeria; b Parasitology and Epidemiology Unit, Department of Animal and Environmental Biology, Federal University Oye-Ekiti, Nigeria; c International Center of Photography, New York, USA; d Smiley Optical Services Limited, Abuja, Nigeria; e Neglected Tropical Disease Unit, State Ministry of Health, Taraba, Nigeria; f Adrian Hopkins Consulting, England, United Kingdom

**Keywords:** Onchocerciasis, Blindness, Fundus camera, Elimination, Nigeria, Taraba

## Abstract

**Introduction:**

Onchocerciasis is the world's second leading cause of infectious blindness and remains a major problem in parts of Africa. In light of the efforts targeted towards improving ongoing elimination program, this study assessed onchocerca-induced visual impairments in Gashaka local government areas (LGA) in Taraba State, north-eastern Nigeria.

**Methods:**

In 2019, we recruited 158 consenting visually impaired persons across three communities in Garbabi ward of Gashaka LGA. To avoid confusion with co-endemic trachoma, the integrity of the tarsal conjunctiva, eyelashes were assessed using direct light. The anterior segment of the eye was also examined using a torchlight with oblique illumination. However, the posterior segment of the eye was assessed using a fundus camera. Two photographic images for the left and right eye of each participant were captured using the clinical fundus camera. The photographic eye images that were too dark were discarded, and only clear images were analyzed by two ophthalmologists. An ocular manifestation report was recorded for each participant following consensus between the ophthalmologists.

**Results:**

Of the 316 photographic eye images, almost half 146 (46.2%) from 73 participants were just too destroyed for light to penetrate and was not included in the analysis. Only 170 from 85 participants were clear and examinable. A total of 33 (39%) participants had chorioretinitis suggestive of onchocerciasis, including 22(25.9%) with chorio-retinal atrophy, 7(8.2%) and 4(4.7%) had chorioretinal atrophy in combination with early cataract and signs of trachoma respectively. In addition, 3(3.5%) of the participant had eye images which showed lens opacities, 1(1.2%) showed signs of keratoconus and 1(1.2%) showed a scared and pigmented cornea, possibly due to onchocerciasis. Furthermore, 28 (32.9%) had some ill-defined changes and 19 (22.4%) showed poorly defined chorio-retinal atrophy.

**Conclusion:**

In a bid to sustain MDA gains towards elimination of onchocerciasis, this work highlights the need for continuous assessment of onchocerciasis induced visual impairment, strengthening of ivermectin delivery and optimizing compliance and patient care among affected populations. These would require resource acquisition and local capacity building. Our preliminary findings call for further operational research on ocular morbidity as well as future stakeholders' consultations in this important and understudied area.

## 1 Introduction

Onchocerciasis has been characterized by the World Health Organization as a neglected tropical disease because of the chronic, disabling, and disfiguring effect it has on the skin and eye of extremely poor and neglected populations (Hotez and Kamath, 2009). The disease which is also known as river blindness is the world's second leading cause of infectious blindness, and is caused by the parasitic filarial worm *Onchocerca volvulus*. The parasites are transmitted by bites of infected blackflies (*Simulium spp*), that breed along fast-flowing rivers and streams, close to remote villages located near fertile land where people rely on agriculture (World Health Organization, n.d.). Onchocerciasis has been an important public health problem, most predominantly in Africa, some parts of Latin America and Yemen. About 99% of the estimated 37 million estimated cases reside in 31 African countries (World Health Organization, n.d.; Zouré et al., 2014; Amazigo et al., 2006; Hotez et al., 2007), with a wide geographic distribution extending from Senegal in the West to Ethiopia in the East and from Mali in the north to Angola and Malawi in the south (Boatin and Richards, 2006).

The clinical manifestations in the skin and eye of infected persons are associated with inflammatory responses caused by the dying (larvae) microfilariae (World Health Organization, n.d.). This inflammatory response causes severe itching, changes in skin colour and loss of skin elasticity (Centre for Disease Control (CDC), n.d.). Ocular changes can be found in any part of the eye, and are characterized based on the location of the damage, as posterior or anterior eye disease (Hall and Pearlman, 1999; Etya'ale, 2001). Anterior segment disease is caused by dying filarial worms in the cornea or anterior chamber of the eye. These worms induce inflammatory responses which causes corneal opacification (punctate keratitis). Continued exposure to the parasites or heavy infection, and without treatment may lead to complete scarring of the cornea (sclerosing keratitis) with resultant blindness (Hall and Pearlman, 1999; Abiose, 1998; Pearlman and Lass, 1994). The disease can also cause chronic uveitis, secondary glaucoma and cataract. Inflammation in the posterior segment include atrophy of the retinal pigment epithelium (Abiose, 1998), and may become worsened by subretinal fibrosis. It is believed that posterior eye disease is induced by autoimmune responses, and cross-reactive proteins as a result of the *Onchocerca* parasite (Chan et al., 1987; McKechnie et al., 1993; Semba et al., 1990; Van der Lelij et al., 1990).

Ocular manifestations are more prevalent in the savannah regions of West Africa, whereas blindness is rare in persons infected in the rain forest regions (Dadzie et al., 1990; Dadzie et al., 1989). Efforts targeted at controlling Onchocerciasis in the savannah areas of West Africa commenced in 1975, with blackfly control by the Onchocerciasis Control Program (OCP) (Amazigo et al., 2006), and with the availability of ivermectin (Mectizan ®) control expanded to other endemic communities from the early 1990s and became the chief strategy of the African Programme for Onchocerciasis Control (APOC) established in 1995 in non OCP countries (Amazigo et al., 2006; African Programme for Onchocerciasis Control (WHOAPOC), 2010). Following the closure of APOC in 2015, the Expanded Special Project for Elimination of Neglected Tropical Diseases (ESPEN) was established in 2016 to maintain the gains made over the past two decades by integrating this approach across the five neglected tropical diseases in Africa, including onchocerciasis elimination (WHO Regional Office for Africa (AFRO), n.d.; Hopkins, 2016).

In Nigeria, the National Onchocerciasis Control Programme (NOCP) was established in 1987, and commenced massive distribution (MDA) of ivermectin in 1991 using the community-directed treatment approaches. Over the past 3 decades, a large international and local partnership has successfully assisted in the effort towards elimination of onchocerciasis (Amazigo et al., 2006; African Programme for Onchocerciasis Control (WHO/APOC), n.d.; *Federal Ministry of Health (FMoH*), 2017; Olamiju et al., 2021). Taraba, one of states in the north-eastern part of Nigeria, was classified to be hyper-endemic and a priority area for MDA in Nigeria (*Federal Ministry of Health (FMoH*), 2017). The state has benefitted from over 23 rounds of MDA since 1997. In 2015, the state was reclassified with a suspected transmission interruption status by the Federal Ministry of Health and National Onchocerciasis Elimination Committee (NOEC) (Griswold et al., 2018). It is therefore important to conduct intermittent surveillance and spot-check assessments in highly endemic areas to monitor recrudescence, with the aim of informing programmatic actions, in preparation for assessment and verification of elimination status. Because of the high prevalence of blindness in the community visually impaired persons living in the Gashaka area of Taraba State north eastern Nigeria were assessed to confirm if infection with *Onchocerca volvulus* was still a major cause of visual impairment.

## 2 Materials and methods

### 2.1 Study area

**Fig. 1:**
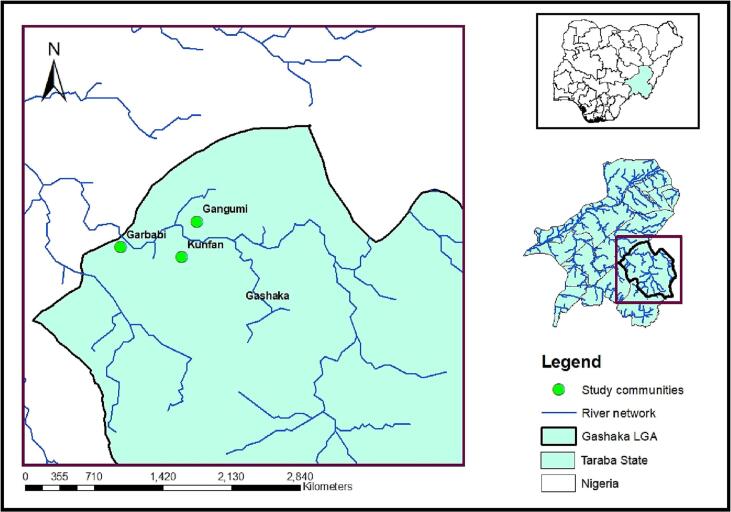
Map of Taraba State showing the study LGA.

This study was conducted in Gashaka, one of the 16 administrative LGAs in Taraba state (Fig. 1). The LGA is hyper-endemic for onchocerciasis with an estimated prevalence of 41.8% and a considerable number of visually impaired persons (Federal Ministry of Health (FMoH), 2021). The Onchocerciasis control unit was established in 1994 and later expanded in 2016 to the Neglected Tropical Diseases (NTDs) control department that currently oversees the morbidity control activities of onchocerciasis and other major NTDs. The mode of operation involves a chain of stakeholders at different administrative levels including, frontline health workers at primary health care centres, community leaders and drug distributors. The LGA have successfully implemented 24 rounds of mass drug administration, with 23 effective rounds (>65%) recorded (Federal Ministry of Health (FMoH), 2021).

### 2.2 Ethical considerations

The study protocol received approval from the State Ministry of Health. Approvals and field permits were also obtained from the NTD control unit and the primary health care coordinator in the LGA. Standardized consent statements and objectives of the study were read in the local languages to the village head by survey teams. Written informed consents were also obtained from eligible participants prior to collection of demographic information such as age and sex. The team leader informed participants of the study protocol as regards strict compliance to confidentiality and anonymity of participant's data. Parents of minors (children below 16 years of age) also provided written consents in addition to the assent provided by the consenting children to be recruited. Participation was completely voluntary and no incentives were offered to lure participants into the study.

### 2.3 Study design and election of communities and participants

This study was cross-sectional in design involving purposive selection of three study communities; Garbabi, Kunfan and Gangumi based on; (1) high number of visually impaired persons, and (2) proximity to the only blind school in Taraba State. A total sampling method was employed involving invitation of all visually impaired persons in each of the study communities. Communities were visited and sensitized about the study procedures through the community leader and drug distributors. Eligible members were further invited through the use of town announcers. Inclusion criteria were; (1) members who were above age 5, (2) sleeps in the community for at-least 3 nights every week, and (3) are visually impaired. In each community, a site, usually at the centre of the community, or as suggested by the community leader, was designated as an area of work.

### 2.4 Assessment of participants' eye

A non-identifier study number was assigned to each participant’ after consents had been taken with the help of the caregiver. The integrity of the tarsal conjunctiva, eyelashes and cornea were first assessed for scaring and opacity using a torchlight with oblique illumination. Afterwards, the retina was assessed using the clinical non-mydriatic fundus camera (Model: Canon CR-DGi NM camera) by a trained technician after dilatation of the pupil.

### 2.5 Analysis of the photographic eye image from the clinical fundus camera

Two photographic images for the left and right eye of each participant were captured using the clinical fundus camera. The photographic eye images were analyzed by two ophthalmologists, and after a consensus was reached, an ocular manifestation report was recorded for each participant. The photographic eye images that were too dark were discarded and not included in the final analysis.

### 2.6 Data analysis

Data analysis was performed in SPSS 25.0. The demographic data of the participants were analyzed using descriptive statistics such as frequencies, percentages and cross tabulation. Arc GIS 9.3 was used for map creation.

## 3 Results

### 3.1 Characteristics of study participants

A total of 158 participants consented; with the majority 124(78.5%) from Garbabi, 20 (12.7%) from Gangumi and 14 (8.9%) from Kunfan. The majority of the respondents were males 105(66.5%) and 53(33.5%) were females. There were no significant differences in the proportion of participants recruited across the study communities and their gender (*p* > 0.05). The majority of the participants in Garbabi were within the age-range 16 and 45 years, compared to participants in Gangumi and Kunfan, who were above 46 years. There were therefore significant differences in the proportion of participants recruited across the study communities and age categories (*p* < 0.05) (Table 1).

**Table 1:** Characteristics of study participants.

	Communities				
	Gangumi	Garbabi	Kunfan	Total	*p*-value
**Sex**					
Male	13 (65.0)	80 (64.5)	12 (85.7)	105 (66.5)	0.278
Female	7 (35.0)	44 (35.5)	2 (14.3)	53 (33.5)	
Total	20 (12.7)	124 (78.5)	14 (8.9)	158 (100)	

**Age group (in years)**					
5–15	0(0)	27(21.8)	0(0)	27(17.1)	0.000
16–25	1(5.0)	56 (45.2)	0 (0)	57 (36.1)	
26–45	4(20.0)	31(25.0)	1(7.1)	36(22.8)	
46–65	9(45.0)	7 (5.6)	9(64.3)	25 (15.8)	
>65	6 (30.0)	3 (2.4)	4 (28.6)	13 (8.2)	
Total	20 (12.7)	124 (78.5)	14 (8.9)	158 (100)	

### 3.2 Ocular manifestations of the study participants

Of the 316 photographic eye images, almost half 146 (46.2%) from 73 participants were just too destroyed for light to penetrate and therefore was not included in the analysis. Only 170 from 85 participants were clear and examinable. A total of 33 (39%) participants had chorioretinitis suggestive of onchocerciasis, including 22(25.9%) with chorio-retinal atrophy, 7(8.2%) and 4(4.7%) had chorioretinal atrophy in combination with early cataract and signs of trachoma respectively. In addition, 3(3.5%) of the participant had eye images which showed lens opacities, 1(1.2%) showed signs of keratoconus and 1(1.2%) showed a scared and pigmented cornea, possibly due to onchocerciasis. Furthermore, 28 (32.9%) had some ill-defined changes and 19 (22.4%) showed poorly defined chorio-retinal atrophy (Table 2).

**Table 2:** Ocular manifestations of study participants.

Ocular manifestation	N (%)	Cause	Assertiveness score (0,1)
Chorio-retinal atrophy	22 (25.9)	Onchocerciasis	1
Poorly defined chorio-retinal atrophy	19 (22.4)	Probability of Onchocerciasis	0
Chorio-retinal atrophy with less dense lens opacity	7 (8.2)	Cataract and Onchocerciasis	1
Lens opacity	3 (3.5)	Cataract	1
Chorio-retinal atrophy together with eyelid signs of old trachoma	4 (4.7)	Probable onchocerciasis and possible old trachoma	1
Ill-defined changes	28 (32.9)	Not defined	0
Keratoconus	1 (1.2)	Keratoconus	1
Probable sclerosing keratitis	1 (1.2)	Cornea damage	1
	**85 (100)**		

N: Number of participants with ocular manifestations.

## 4 Discussion

Following the 2030 neglected tropical diseases road map, onchocerciasis is targeted for elimination in at least 25 countries (World Health Organization, 2021). In Nigeria, transmission has only been interrupted in 2 of the 36 states including the Federal Capital Territory, after successful implementation of annual ivermectin mass drug administration for a period of 8–26 years (Richards et al., 2020). Taraba state has also benefitted from uninterrupted and successful rounds of ivermectin mass drug administration for the past 26 years. It has therefore been speculated that the state might have reached elimination stage, based on the recommendations of Winnen (Winnen et al., 2002), whose models showed that hyperendemic communities would need about 27 rounds of ivermectin MDA before reaching the elimination milestone.

While interruption of transmission depends on parasitological and entomological data, in view of the debilitating and disabling nature of onchocerciasis, most especially on the eyes of affected persons, it is important to identify persons with visual impairment with the aim of providing supportive care (TY Danjuma Foundation (TYDF), 2020). Direct ophthalmoscopy which involves using a hand-held powered device with a series of lenses and light source has been employed for examination of patients' eye for diabetic retinopathy, cataract, and glaucoma (Carlsson et al., 2006). However, direct ophthalmoscopy requires extensive practice to accurately recognize optic nerve and retinal abnormalities and often requires pupil dilatation. Recent studies have suggested that digital retinal photography can replace direct ophthalmoscopy in many settings (Pérez et al., 2012). Our study therefore utilized the clinical fundus camera to assess the retina and choroid of study participants for onchocerca-induced changes. Almost half of the eyes inspected were not examinable because light couldnot penetrate, and about 40% of the examined eyes had posterior segment eye disease with choroid and retina damage, indicative of onchocerciasis. This finding suggests the predominance of onchocerca-induced visual impairment over other eye conditions in the population studied.

Public health interventions such as direct assessment of the eye, surgical operations and administration of antibiotics are available to reverse early-stage blindness induced by trachoma and cataract (TY Danjuma Foundation (TYDF), 2020; Umar et al., 2018). However, the direct assessment of the eye of persons infected with onchocerciasis for holistic programme planning have gained limited attention till date, as there are no treatment modalities once the irreversible stages of blindness have been reached. As such the risk of developing onchocerca-induced skin or posterior eye disease can only be mitigated when compliance to ivermectin mass drug administration is optimal (Abiose, 1998; Pearlman and Lass, 1994; Chan et al., 1987; McKechnie et al., 1993; Semba et al., 1990). Taraba state has been one of the endemic foci of Onchocerciasis in Nigeria with a high prevalence of blindness and other complications associated with Onchocerciasis. Endemic communities in the state have been on ivermectin since 1995. It is envisaged that long term chemo-therapeutic intervention should, with time and continuous good coverage (>65%), protect the affected individuals from developing blindness and severe skin manifestation via reduced transmission. While onchocerciasis control in much of Africa is indisputably a major public health achievement, evidence of suspected interrupted transmission in Taraba and several other foci in Nigeria raises hopes for eventual elimination. It is important to sustain the gains of this intervention to avoid recrudescence of transmission (The Carter Center, 2014; Olamiju et al., 2014). Our findings therefore suggest that; (1) identifying visually impaired persons through investment in direct ophthalmoscopy will help to identify patients who need extra support, and (2) developing strategies to strengthen ivermectin delivery and ensuring compliance among these populations is of vital importance. These actions would serve as a as a step in the right direction to improve patient care and avoid recrudescence of transmission of Onchocerciasis and ultimately help sustain MDA gains in view of the elimination targets set for 2030.

## 5 Conclusion

In a bid to sustain MDA gains, this work highlights the need for; (1) routine assessments of onchocerciasis induced visual impairment for patient management in countries and foci where ocular manifestations is endemic, as well as (2) strengthening the delivery of ivermectin. Inclusion of ocular screening in routine programming efforts would require acquisition of suitable camera for digital retinal photography and local capacity building on the use of the camera and interpretation of recorded images. We therefore hope our preliminary findings will stimulate further operational research as well as future stakeholders' consultations in this important and understudied area.

## Consent for publication

Not applicable.

## Availability of data and materials

There are legal restrictions on sharing the dataset, as they are owned by a third-party. Also, as part of the ethical guidelines, there are imposed restrictions on sharing this data except when necessary and a request for data sharing must be filed. As such, request for the dataset can be sent to Neglected Tropical Disease Unit, Taraba State Ministry of Health, Nigeria.

## Funding

Not Applicable.

## Authors' contribution

FOO conceptualized and designed the study. HOM, DKI, AJM, OJO, AH improved the study protocol. MTB, VO, MN, DKI participated in field and ophthalmological assessment. HOM performed statistical analysis and prepared the first draft of the manuscript. FOO, HOM, MTB, VO, MN, AJM, OJO, AH, DKI contributed to the development of the final manuscript and approved its submission.

## Declaration of Competing Interest

The authors declare that they have no competing interest.
